# Safety of Simultaneous Hepatectomy and Splenectomy in the Treatment of Hepatocellular Carcinoma Complicated with Hypersplenism: A Meta-analysis

**DOI:** 10.1155/2019/9065845

**Published:** 2019-08-14

**Authors:** Xuefeng Liu, Zhiqiang Chen, Meng Yu, Wei Zhou, Xuting Zhi, Tao Li

**Affiliations:** Department of General Surgery, Qilu Hospital of Shandong University, Jinan, Shandong, China

## Abstract

**Background:**

We conducted this meta-analysis to compare the efficacy and safety of simultaneous hepatectomy and splenectomy (HS) with hepatectomy alone (HA) in patients with hepatocellular carcinoma (HCC) and hypersplenism.

**Materials and Methods:**

A systematic search was conducted in PubMed, Embase, Cochrane Library, and Wanfang Data through March 1, 2018, with no limits. Two investigators independently screened all retrieved studies. The investigators of the original publications were contacted if required information was absent. All the included studies were managed by EndNote X7. Quality assessment of the included studies was performed using a modified Newcastle-Ottawa Scale judgment. Extracted data for each endpoint were analyzed by using STATA 12.0 software.

**Results:**

Thirteen studies, including a total of 1468 patients, comparing the effects of HS with HA were pooled in this meta-analysis. Outcomes including postoperative complications, perioperative mortality, intraoperative blood transfusion, and albumin (ALB) content at postoperation day (POD) 7 did not differ significantly between the two groups. Simultaneous approaches significantly promoted 1-, 3-, and 5-year disease-free survival (DFS) rates and overall survival (OS) rates, prolonged operation time, aggravated intraoperative blood loss, increased white blood cell (WBC) and platelet (PLT) counts at POD 7, and lowered total bilirubin (T-Bil) contents at POD 1 and 7.

**Conclusion:**

Compared to HA, HS is safer and more effective in ameliorating liver function and improving survival of HCC patients complicated with hypersplenism. This trial is registered with CRD42018093779.

## 1. Introduction

Hepatocellular carcinoma (HCC) is the sixth most common neoplasm and the third leading cause of cancer death [1, 2]. Though many kinds of therapeutic strategies are available for HCC, hepatectomy is still the first-line treatment [3]. However, more than 85% of HCC patients in Asia are complicated with liver cirrhosis [3, 4], and the high proportion of coexistent hypersplenism among cirrhotic patients will cause secondary thrombocytopenia, hyperbilirubinemia, and immunosuppression [5]. For HCC patients with decompensated liver cirrhosis, hepatectomy is considered to be contraindicated [6], in spite of the progress of surgical techniques and perioperative supportive therapy. Recently, it is reported that splenectomy may promote postoperative hematological indexes, ameliorate liver function, facilitate liver regeneration, improve immune response, and reduce the HCC risk in cirrhotic patients [7–10]. Moreover, for patients with HCC and hypersplenism, splenectomy is thought to ameliorate survival conditions and allow patients to undertake aggressive but effective therapies [11–14]. Therefore, splenectomy was introduced to combine with hepatectomy to treat HCC patients complicated with hypersplenism. But the rationality of simultaneous hepatectomy and splenectomy (HS) is hitherto controversial. Previously, Li et al. [15] conducted a meta-analysis in 2015 to compare HS with hepatectomy alone (HA). But they only pooled 8 studies with limited outcomes and deficient outcome data. Three years have gone, and more studies have been published. We believed that it was necessary to update the meta-analysis and introduce more outcomes to further illustrate the efficacy and safety of HS in treating patients with HCC and hypersplenism.

## 2. Patients and Methods

### 2.1. Selection of Trials

We conducted a systematic search in PubMed, Embase, Cochrane Library, and Wanfang Data through March 1, 2018, with no limits. The search strategies were based on combinations of the following key words: hepatocellular carcinoma, liver cancer, portal hypertension, hypersplenism, liver cirrhosis, liver fibrosis, thrombocytopenia, liver resection, hepatectomy, and splenectomy. The computer search was supplemented with manual searches in the reference lists of all retrieved review articles, primary studies, and abstracts from meetings to identify other studies beyond it. When the results of a single study were reported in more than one publication, only the most recent and more comprehensive data were included in the meta-analysis.

Studies were included if (1) they had a clear diagnosis of HCC (including computed tomography, magnetic resonance imaging, serum alpha-fetoprotein levels, and pathology after surgery), splenomegaly (splenic thickness of more than 4.1 cm on transcutaneous ultrasonography or computed tomography), and hypersplenism (WBC < 3.5 × 10^9^/l or PLT < 100 × 10^9^/l); (2) they were randomized control trials (RCTs), cohort studies, or case-control studies comparing HS with HA and had available data for each of the surgical approaches; and (3) they reported sufficient data for outcomes, including survival data (disease-free survival (DFS) and overall survival (OS) rates), operation-related data (operation time, intraoperative blood loss, intraoperative blood transfusion, postoperative complications, and perioperative mortality), hematological data (WBC and PLT counts at postoperative days (POD) 1, 7, and 30), and liver function indicators (alanine transaminase (ALT), aspartate transaminase (AST), total bilirubin (T-Bil), and albumin (ALB) contents at POD 1, 7, and 30, respectively).

Studies were excluded if (1) they were animal studies or in vitro studies, (2) they only reported one surgical procedure (hepatectomy or splenectomy), (3) they compared HS with other surgical approaches, such as simultaneous hepatectomy and splenic artery embolization, (4) data could not be used for statistical analysis, (5) hepatectomy and splenectomy were conducted step by step, (6) baseline characteristics of the two groups were significantly incomparable, and (7) articles from the same author or institution contained significant overlap in patient data.

The screening of retrieved studies was completed by two investigators independently, and disagreements were solved through discussion or consulting a third party.

This meta-analysis was registered in PROSPERO, and the registration number is CRD42018093779.

### 2.2. Data Extraction and Quality Assessment

Data on all random variables and targeted outcomes were extracted from eligible studies by two reviewers independently. The extracted information included baseline information of articles (authors, research areas, and publication year), general information (case numbers, mean age, sex ratio, Child-Pugh classification, tumor number, type of hepatectomy (major or minor hepatectomy)), and treatment outcomes (DFS and OS rates, operation time, intraoperative blood loss, intraoperative blood transfusion, postoperative complications, perioperative mortality, WBC and PLT counts at POD 1, 7, and 30, respectively, along with ALT, AST, T-Bil, and ALB contents at POD 1, 7, and 30, respectively). Disagreements were resolved by discussion or consulting experts. If necessary, the primary authors were contacted to obtain missing data. A modification of the Newcastle-Ottawa Scale was used as an assessment tool for selection, comparability, and outcome assessment.

### 2.3. Outcome Definition

Perioperative mortality was defined as death in the hospital within 30 days following surgery. Complications included both hepatic and extrahepatic events. Major hepatectomy was defined as resection of three or more segments, while minor hepatectomy was the opposite.

### 2.4. Statistical Analysis

Extracted data for each endpoint were analyzed by using STATA 12.0 software. We analyzed binary variables using risk ratios (RRs) along with 95% confidence intervals (CIs) and analyzed continuous data using standard mean differences (SMDs) along with 95% CIs. The *I*^2^ and *P* value were used for evaluation of heterogeneity. A fixed-effects model (fixed, Mantel-Haenszel for binary variables; fixed, inverse variance for continuous variables) was used when the heterogeneity test showed better homogeneity (*P* > 0.1, *I*^2^ ≤ 50%). Otherwise, a random-effects model (random (M-H heterogeneity) for binary variables; random (I-V heterogeneity) for continuous variables) was used. And if significant heterogeneity was found among studies, we conducted subgroup analysis, univariate logistic regression analysis, and sensitivity analysis (by omitting each single study) to figure out its origin. With respect to WBC and PLT counts at POD 1 and 30, ALT and AST counts at POD 1, 7, and 30, T-Bil content at POD 30, and ALB content at POD 1 and 30, studies available were deficient, so we quit conducting meta-analysis for those outcomes.

We used six stratifying variables: publication year (published before or after 2010), study location, etiology (complicated with HBV only or HBC and HCV), sex ratio (proportion of male was more than 0.5 or not), Child-Pugh classification (proportion of patients whose Child-Pugh classification was A was less than 0.75 or not), type of hepatectomy (proportion of major hepatectomy (resection of three or more hepatic segments) was more than 0.3 or not), intraoperative blood loss (more than 800 ml or not), and intraoperative transfusion (proportion of patients receiving intraoperative blood transfusion was more than 0.5 or not).

Egger's regression asymmetric test was used to examine potential publication bias related to endpoints, including five-year DFS and OS rates, operation time, intraoperative blood loss, intraoperative blood transfusion, postoperative complications, perioperative mortality, and PLT and T-Bil counts at POD 7, all of which were reported in more than five studies. If the test implies significant publication bias, the fail-safe number (*N*_fs0.05_) was calculated to determine what extent the bias influenced reliability of the outcome: *N*_fs0.05_ = (*ΣZ*/1.64)^2^‐*κ*, where *Z* is the *Z* value of each study and *κ* is the number of included studies [16]. The larger the *N*_fs0.05_ is, the more reliable the outcome is.

## 3. Results

### 3.1. Characteristics of Pooled Studies

**Figure 1:**
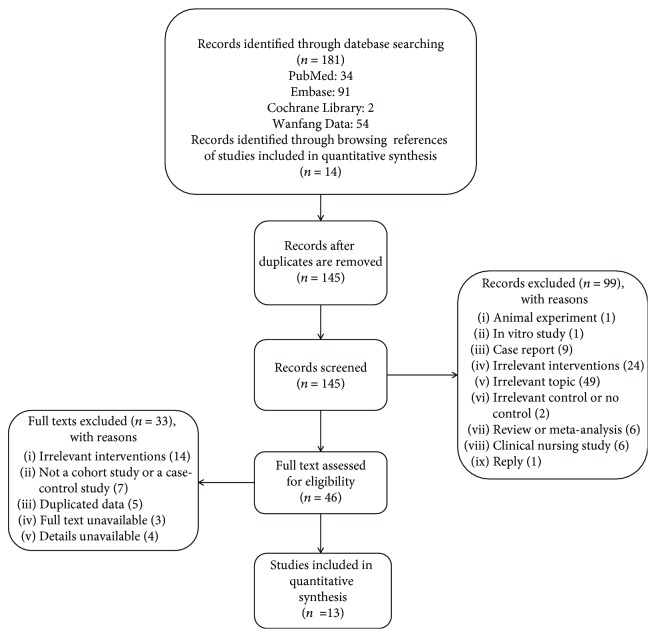
Flow diagram of trial selection.

After 50 duplicated studies had been excluded, we excluded 99 studies by browsing the title and abstract. And by the full text screening, we eventually pooled 13 studies including a total of 1468 patients to compare the effects of HS with HA in this meta-analysis [12, 13, 17–27].  Figure 1 shows more details about our search and inclusion strategy. All studies included are retrospective cohort studies. Among the total of 13 studies included, seven papers were published in Chinese [17, 18, 21–24, 26], and others were in English [12, 13, 19, 20, 25, 27]. Twelve studies were from China [12, 13, 17–24, 26, 27], and one was from Korea [25]. All studies included were uniethnic and analyzed eastern Asian.  Table 1 summarizes the characteristics of included studies.

### 3.2. Quality Judgments for Studies

Qualities of all the included studies were analyzed by using the modified Newcastle-Ottawa Scale, retrospectively. The results of the quality judgments are shown in Table 2.

### 3.3. Survival Data

**Figure 2:**
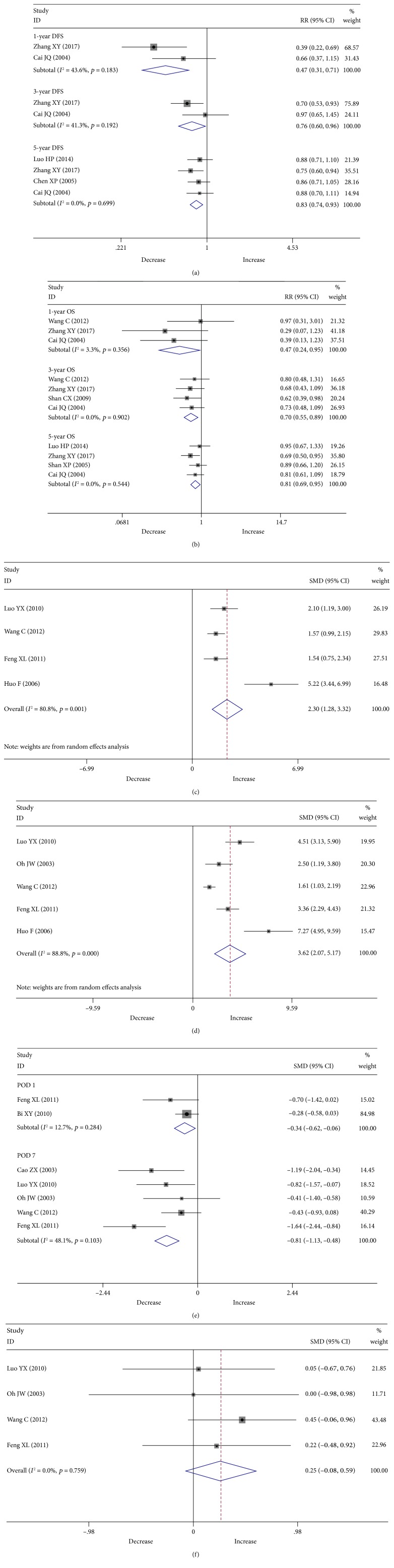
Meta-analysis of comparison between the HS and HA groups for the (a) DFS rate, (b) OS rate, (c) WBC count at POD 7, (d) PLT count at POD 7, (e) T-Bil content at POD 1 and 7, and (f) ALB content at POD 7. Abbreviations: HS: simultaneous hepatectomy and splenectomy; HA: hepatectomy alone; DFS: disease-free survival; OS: overall survival, WBC: white blood cell; PLT: platelet; T-Bil: total bilirubin; ALB: albumin; POD: postoperative day.

With respect to survival data, six endpoints, including 1-, 3-, and 5-year DFS and OS rates, were analyzed (Figure 2). Four studies reported a five-year DFS rate [13, 18, 20, 23]. HS significantly increased the five-year DFS rate compared with HA (RR 0.83, 95% CI 0.74-0.93, *P* = 0.001; Figure 2(a)). Two studies reported both one-year and three-year DFS rates, and they were both significantly higher in the HS group (RR 0.47, 95% CI 0.31-0.71, *P* ≤ 0.001 and RR 0.76, 95% CI 0.60-0.96, *P* = 0.024, respectively; Figure 2(a)) [13, 18]. Four studies reported a five-year OS rate [13, 18, 20, 23]. We found that the five-year OS rate in the HS group was significantly higher than that in the HA group (RR 0.81, 95% CI 0.69-0.95, *P* = 0.011; Figure 2(b)). Four studies reported a three-year OS rate [13, 18, 26, 27]. A higher three-year OS rate was found in the HS group, which was highly significant (RR 0.70, 95% CI 0.55-0.89, *P* = 0.003; Figure 2(b)). Three studies reported a one-year OS rate, which showed that the HS group had a significantly higher one-year OS rate than the HA group (RR 0.47, 95% CI 0.24-0.95, *P* = 0.036; Figure 2(b)) [13, 18, 27].

### 3.4. Hematological Results

With respect to hematological results, two endpoints, including WBC and PLT counts at POD 7, were analyzed, respectively (Figure 2). Four studies reported WBC count at POD 7, which was significantly higher in the HS group (SMD 2.30, 95% CI 1.28-3.32, *P* ≤ 0.001; Figure 2(c)) [21, 22, 24, 27]. Five studies reported PLT counts at POD 7 [21, 22, 24, 25, 27], which was significantly higher in the HS group than in the HA group (SMD 3.62, 95% CI 2.07-5.17, *P* ≤ 0.001; Figure 2(d)).

### 3.5. Liver Function-Related Results

For this section, three endpoints, comprised of T-Bil content at POD 1 and ALB and T-Bil contents at POD 7 (Figure 2), were analyzed. Two studies reported T-Bil content at POD 1 [17, 21]. We found a lower level in the HS group (SMD -0.34, 95% CI -0.62 to -0.06, *P* = 0.017; Figure 2(e)). Five studies reported T-Bil content at POD 7, which was significantly lower in the HS group than in the HA group (SMD -0.81, 95% CI -1.13 to -0.48, *P* ≤ 0.001; Figure 2(e)) [19, 21, 24, 25, 27]. Four studies reported ALB content at POD 7 [21, 24, 25, 27], and no significant differences were found between the two surgical approaches (SMD 0.26, 95% CI -0.08-0.59, *P* = 0.134; Figure 2(f)).

### 3.6. Operation-Related Results

**Figure 3:**
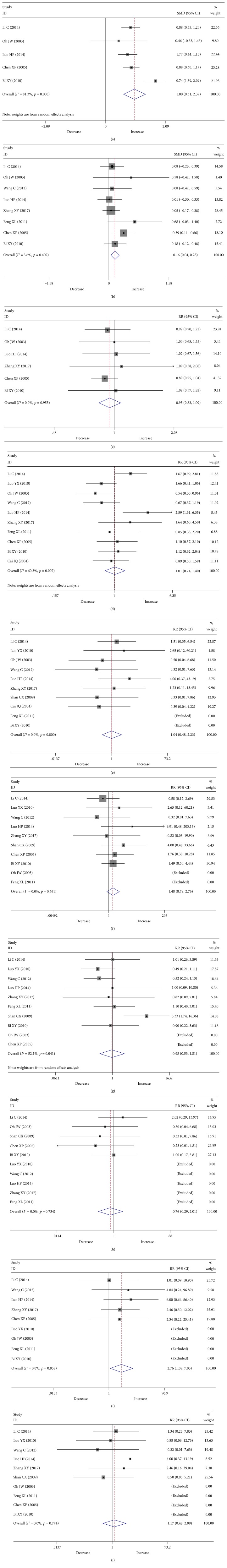
Meta-analysis of comparison between the HS and HA groups for (a) operation time, (b) intraoperative blood loss, (c) intraoperative blood transfusion, (d) postoperative complications, (e) perioperative mortality, (f) infection, (g) ascites, (h) upper gastrointestinal bleeding, (i) abdominal bleeding, and (j) acute liver failure. Abbreviations: HS: simultaneous hepatectomy and splenectomy; HA: hepatectomy alone.

For operation-related results, there are five endpoints—operation time, intraoperative blood loss, intraoperative blood transfusion, postoperative complications, and perioperative mortality (Figure 3). Five studies reported operation time [12, 17, 20, 23, 25]. And as we can see in Figure 3, HS significantly prolonged the operation time (SMD 1.00, 95% CI 0.61-1.39, *P* ≤ 0.001; Figure 3(a)). Eight studies reported intraoperative blood loss [12, 13, 17, 20, 21, 23, 25, 27]. The volume of intraoperative blood loss in the HS group was higher than that in the HA group (SMD 0.16, 95% CI 0.04-0.28, *P* = 0.009; Figure 3(b)). Six studies reported intraoperative blood transfusion, which was not significantly different between the two surgical approaches (RR 0.95, 95% CI 0.83-1.09, *P* = 0.442; Figure 3(c)) [12, 13, 17, 20, 23, 25]. Ten studies reported postoperative complications, which did not differ between the two surgical approaches (RR 1.01, 95% CI 0.74-1.40, *P* = 0.929; Figure 3(d)) [12, 13, 17, 18, 20, 21, 23–25, 27]. Moreover, we conducted meta-analysis for some of the postoperative complications, including infection, ascites, upper gastrointestinal bleeding, abdominal bleeding, and acute liver failure (Figures 3(f)–3(j)). As shown in Figure 3, disparity could be seen only in abdominal bleeding (RR 2.76, 95% CI 1.08-7.05, *P* = 0.034; Figure 3(i)), which was more common in the HS group, while other complications showed no significant difference between the two groups. Ten studies reported perioperative mortality, while two studies were excluded from conducting analysis because no people died perioperatively in both groups. There was no significant difference between the two groups (RR 1.04, 95% CI 0.48-2.23, *P* = 0.922; Figure 3(e)) [12, 13, 17, 18, 21, 23–27].

### 3.7. Heterogeneity

High heterogeneity was detected for operation time (*I*^2^ = 81.3%, *P* ≤ 0.001, Tau^2^ = 0.1491), as well as WBC and PLT counts at POD 7 (*I*^2^ = 80.8%, *P* = 0.001, Tau^2^ = 0.8196 and *I*^2^ = 88.8%, *P* ≤ 0.001, Tau^2^ = 2.6330, respectively). Moderate heterogeneity was detected for postoperative complications (*I*^2^ = 60.3%, *P* = 0.007, Tau^2^ = 0.1559). Heterogeneity results for other endpoints were all acceptable.

Four endpoints, including WBC and PLT counts at POD 30, along with ALT and AST contents at POD 1, which have high or moderate heterogeneity, were difficult to analyze for heterogeneity. For that reason, we quit conducting quantitative analysis for those outcomes.

The heterogeneity results for operation time did not change significantly after conducting subgroup analysis. While the heterogeneity for operation time disappeared by omission, Bi et al. [17] (*I*^2^ = 0.0%, *P* = 0.828) and the effect remained the same (SMD 0.83, 95% CI 0.66-1.01, *P* ≤ 0.001).

To figure out the origin of heterogeneity for WBC count at POD 7, we conducted a subgroup analysis by year, which showed that there was no heterogeneity for studies published after 2010 (*I*^2^ = 0.0%, *P* = 0.584, Tau^2^ = 0.0000). And the pooled estimate of studies after 2010 WBC counts at POD 7 was 1.67 (95% CI 1.26-2.09, *P* ≤ 0.001).

With respect to PLT count at POD 7, neither subgroup analysis nor sensitivity analysis did not eliminate the heterogeneity. Metaregression analysis showed that the sex ratio was associated with these two outcomes, and the proportion of heterogeneity it explained for PLT count at POD 7 was 75.50% (Tau^2^ changed from 2.6330 to 0.6451).

As for the origin of heterogeneity of postoperative complications, by conducting a subgroup analysis by the sex ratio, we found that heterogeneity disappeared in either subgroup. What is more, four studies were included in the subgroup with male proportion > 0.5, and the incidence of postoperative complications in the HS group was significantly higher than that in the HA group (RR 1.64, 95% CI 1.13-2.37, *P* = 0.009), while there was no statistic difference between the two surgical procedures in the subgroup with male proportion ≤ 0.5 (RR 0.76, 95% CI 0.58-1.02, *P* = 0.064).

### 3.8. Publication Bias

Egger's tests for five-year DFS and OS rates, operation time, intraoperative blood loss, intraoperative blood transfusion, postoperative complications, perioperative mortality, and PLT count and T-Bil contents at POD 7 are shown in Table 3, respectively.

As we can see from the tests, there was no statistically significant publication bias for five-year DFS and OS rates, operation time, intraoperative blood loss, intraoperative blood transfusion, postoperative complications, perioperative mortality, or T-Bil contents at POD 7: the *P* value was greater than 0.05 and 95% CI covers 0.

The publication bias for intraoperative blood transfusion and for PLT count at POD 7 was significant (*P* = 0.001, 95% CI 0.548 and 1.105; *P* = 0.021, 95% CI 1.693 and 9.937, respectively). Therefore, *N*_fs0.05_ of these two outcomes was calculated: the *N*_fs0.05_ of intraoperative blood transfusion was -4, which meant that the outcome was quite unsteady, while that of PLT count at POD 7 was 916, which showed that the outcome was pretty reliable.

## 4. Discussion

The high proportion of coexistent cirrhosis among HCC patients restricted hepatic resection [3, 4], and splenomegaly due to increasing portal tension may give rise to secondary hypersplenism, resulting in thrombocytopenia, hyperbilirubinemia, and immunosuppression, all of which may influence the outcome of patients undergoing hepatectomy. Consequently, splenectomy was introduced for those patients, and some retrospective studies indicate that splenectomy might increase the counts of WBC and PLT, ameliorate liver function, facilitate liver regeneration, and raise the chance for HCC patients to receive resection or chemotherapy [7, 8, 10, 28]. In this meta-analysis, we found that HS could significantly increase the counts of WBC and PLT and reduce the T-Bil level, while the ALB levels were comparable with the HA group. For other outcomes, such as hemoglobin (Hb), prothrombin time (PT), and Child-Pugh score, we only conducted qualitative analysis, but previous studies reported that the Hb level between two groups were comparable [27], while the Child-Pugh score was improved in the HS group instead of the HA group [25]. All in all, it is obvious that HS can ameliorate hematological condition and liver function.

Splenectomy preserves liver function from several aspects. Hepatic portal occlusion during hepatectomy will unavoidably bring about ischemia-reperfusion (I/R) injury to multiple organs including the liver [29]. As a consequence, I/R injury triggers the infiltration of multiple inflammatory cells and activates Kupffer cells, which work together by producing inflammatory mediators to give rise to liver damage [30]. Splenectomy can significantly alleviate inflammation in the liver [9, 31], and by eliminating inflammatory cytokines, which arrive in the liver through portal circulation and inhibit liver regeneration, splenectomy plays an important role in ameliorating liver fibrosis and facilitating liver regeneration and then promotes liver function [32–34].

In addition, by reducing portal venous pressure, splenectomy may increase hepatic artery blood flow, which would protect liver function to some degree [29]. Moreover, restriction of endotoxin-induced bacterial translocation caused by splenectomy plays an important role in promoting liver function after hepatectomy as well [35]. Besides, by eliminating spleen-derived endothelin-1 (ET-1), sequentially increasing peripheral nitric oxide (NO) concentration and decreasing the hepatic NO level, splenectomy improves not only intrahepatic portal vein resistance but also splanchnic and systemic hyperdynamic circulation in cirrhotic patients [36], resulting in amelioration in general condition and liver function. Interestingly, Abe et al. [37] discovered that through preventing platelet accumulation by anti-platelet antibody, hepatic protein synthesis was significantly impaired, which suggests that PLTs also contribute to liver growth and regeneration. It is thus evident that the increase of PLT after splenectomy may also contribute to liver function improvement.

When it comes to operative procedures, we might easily associate higher risk with simultaneous approaches, for more extensive surgical trauma, longer operation time, and larger hemorrhage volume. It seems that hepatectomy combined with splenectomy might exacerbate perioperative mortality and postoperative complications. However, our meta-analysis revealed that there was no significant difference between the HS and HA groups for both perioperative mortality and overall postoperative complications. Moreover, incidences of severe complications such as upper gastrointestinal bleeding and acute liver failure are comparable between the HS and HA groups. With respect to other complications, for instance, infection and ascites, HA did not increase the incidences, either.

Major or aggressively extended hepatectomy for liver cancer may give rise to secondary portal hypertension (PH), which would arouse massive ascites, edema, and refractory hemorrhage [38]. Kamanaka et al. [36] found significantly lower blood flow of portal vein and congestion index in cirrhotic patients undergoing splenectomy, which was associated with lower ET-1 and increased NO and contributed to amelioration of complications related to secondary PH such as ascites and upper gastrointestinal bleeding. Similarly, it has been demonstrated that splenectomy improves portal hypertensive gastropathy (PHG) and lowers the incidence of bleeding complication in selected HCC patients, thereby increasing the safety of hepatectomy [39, 40].

Though none of the included studies reported significant discrepancy between the HS and HA groups, we found more vulnerability to abdominal bleeding in patients receiving simultaneous hepatectomy and splenectomy [12, 13, 20, 23, 27]. It is understandable from the more complex surgical procedure and the more massive surgical trauma. Though postoperative reactive hemorrhage more generally occurs from the splenic vessels at the tail of the pancreas, the short gastric vessels, and the trocar sites, which may result in high morbidity and mortality [41], it is avoidable by careful operation and routine inspection of the operative field after removal of the specimen.

Another postoperative complication that receives a lot of attention is portal vein thrombosis (PVT), whose rate following hepatectomy is 9.1%, while that after splenectomy ranges from 0.19 to 17.8% [42, 43]. The diameter of the splenic vein, low WBC counts, and spleen volume are reported as independent risk factors for PVT [7, 44, 45]. In our meta-analysis, PVT was rarely reported by researchers, which made it difficult to conduct quantitative analysis. Kim et al. [11] and Li et al. [12] reported a significantly higher rate of PVT in the HS group than in the HA group. However, the vast majority of those who developed PVT after combined surgery were reported to enjoy complete recovery after standard anticoagulation therapy. Combination of stagnation in the remnant splenic vein, rise of postoperative WBC and PLT counts, and drop of portal vein pressure contribute to formation of PVT [17, 46]. It has been reported that the interphase between splenectomy and symptomatic portal vein thrombosis was 8-12 days [47]. To detect PVT early, computed tomography should be implemented about seven days after splenectomy, and anticoagulation therapy with a low dose of warfarin three to four days after the surgery would be a preferable choice, on the condition of no postoperative hemorrhage [7].

In addition, splenectomy is sometimes associated with infectious complications, especially overwhelming postsplenectomy infection (OPSI), with the life-time incidence of 0.1-0.5% and mortality over 50% [48]. However, our meta-analysis revealed a totally different result that incidences of postsplenectomy infection in two HS and HA groups are comparable, and no OPSI was reported, the reason may be that all patients who underwent splenectomy are adults, which is not the risk factor for the OPSI.

Because of the improvement of perioperative conditions brought by splenectomy, simultaneous approaches do not augment perioperative deaths. We can conclude from this meta-analysis that splenectomy is pretty safe if combined with hepatectomy in the treatment of HCC patients complicated with hypersplenism. For patients who cannot stand simultaneous splenectomy and hepatectomy, splenic artery ligation may be an alternative choice. Though not as effective as splenectomy in improving the liver regeneration [49, 50], splenic artery ligation can be still effective in the treatment of HCC with hypersplenism. It can promote the recovery of liver function, prolong the survival time, and improve the quality of life in HCC patients complicated with liver cirrhosis and hypersplenism [49, 50].

Another conclusion we made was that simultaneous hepatectomy with splenectomy significantly improves survival outcomes of targeted individuals, including DFS and OS rates. Beside the above-mentioned alleviation of inflammatory filtration of the liver, Cao et al. [19] and Chen et al. [20] reported remarkable improvement of antitumor immunity after simultaneous hepatectomy and splenectomy; namely, a higher postoperative CD4+/CD8+ T cell ratio was found in the HS than in the HA group. The accumulation of suppressive macrophages in the spleen will shift the T cell receptor structure and suppress T cell immune function [51], while splenectomy can increase NK cells, which in turn contributes to the recovery of T lymphocyte subsets [19]. As a result, HCC recurrence is reduced in patients undertaking simultaneous hepatectomy and splenectomy. Also, the improved OS rate is associated with promotion of liver function after splenectomy [11]. Moreover, by reducing portal vein pressure and improving portal hypertensive gastropathy (PHG), simultaneous splenectomy significantly decreases the long-term risk of bleeding [26, 39]. More importantly, WBC count increases after splenectomy, which offers favorable conditions for other treatments, such as transcatheter arterial chemoembolization and adjuvant chemotherapy [27]. All of the above contributes to improvement of the OS rate.

This meta-analysis has several limitations. Firstly, all the studies pooled are retrospective and observational. Secondly, the number of patients included in this meta-analysis is small. Thirdly, we only included studies written in languages of English and Chinese. Fourthly, some parameters were absent, which might be potential biases. Fifthly, the qualities of studies published in Chinese might be relatively lower when compared to those in English. Sixthly, there are different degrees of heterogeneities for several outcomes. However, some of them were eliminated by using sensitivity analysis, while others could be partly explained through metaregression analysis. Lastly, Egger's test suggested publication biases for intraoperative blood transfusion and PLT count at POD 7, whereas the latter is quite reliable.

## 5. Conclusion

We have identified that simultaneous hepatectomy and splenectomy increase the postoperative WBC and PLT counts, ameliorates liver function, and improves DFS and OS rates, without increasing postoperative complications and perioperative mortality. All in all, simultaneous hepatectomy and splenectomy is safer, more feasible for patients with HCC and hypersplenism, compared to hepatectomy alone.

## Figures and Tables

**Table 1:** Basic characteristics of all studies pooled in the meta-analysis.

Study	Year	County	Groups	No. of patients (*n*)	No. of male (*n*)	Mean age	Child-Pugh classification			Tumor number		Type of hepatectomy	
							A	B	C	Solitary	Multiple	Minor^†^	Major^‡^
Cao et al. [19]	2003	China	HS	11	11	46	6	5	0	NA	NA	NA	NA
			HA	15	14	41	7	8	0	NA	NA	NA	NA
Li et al. [12]	2014	China	HS	60	46	55.2	52	8	0	47	13	49	11
			HA	121	100	55.8	107	14	0	102	19	95	26
Luo et al. [24]	2010	China	HS	16	NA	NA	14	2	0	NA	NA	NA	NA
			HA	14	NA	NA	10	4	0	NA	NA	NA	NA
Oh et al. [25]	2003	Korea	HS	12	9	48.8	6	6	0	NA	NA	7	5
			HA	6	4	58.7	4	2	0	NA	NA	3	3
Wang et al. [27]	2012	China	HS	31	25	48.94	26	5	0	NA	NA	NA	NA
			HA	30	24	52.33	25	5	0	NA	NA	NA	NA
Luo et al. [23]	2014	China	HS	57	49	49.7	51	6	0	44	13	41	10
			HA	114	106	49.5	105	9	0	94	20	91	20
Zhang et al. [13]	2017	China	HS	110	91	50.19	NA	NA	NA	100	10	99	11
			HA	271	229	49.99	NA	NA	NA	239	32	227	44
Feng et al. [21]	2011	China	HS	12	8	53.12	9	3	0	11	1	NA	NA
			HA	23	17	51.32	19	4	0	22	1	NA	NA
Shan et al. [26]	2009	China	HS	29	26	47.24	15	12	1	25	4	NA	NA
			HA	29	28	53.21	28	0	0	22	7	NA	NA
Huo et al. [22]	2006	China	HS	17	NA	NA	NA	NA	NA	NA	NA	NA	NA
			HA	7	NA	NA	NA	NA	NA	NA	NA	NA	NA
Chen et al. [20]	2005	China	HS	94	80	44.6	64	30	0	75	19	90	4
			HA	110	89	41.7	61	49	0	82	28	103	7
Bi et al. [17]	2010	China	HS	71	59	54	65	6	0	NA	NA	52	19
			HA	106	91	57	96	10	0	NA	NA	82	24
Cai et al. [18]	2004	China	HS	57	40	55.6	48	9	0	NA	NA	45	12
			HA	45	34	50.8	42	3	0	NA	NA	36	9

^†^Minor hepatectomy = irregular hepatectomy and resection of one or two hepatic segments; ^‡^major hepatectomy = resection of three or more hepatic segments. Abbreviations: HS: simultaneous hepatectomy and splenectomy; HA: hepatectomy alone; NA: not available.

**Table 2:** Quality assessment of studies pooled in the meta-analysis based on the modified Newcastle-Ottawa Scale judgment.

Study	Selection^†^	Comparability^‡^	Outcome assessment^§^	Quality judgment
Cao et al. [19]	∗∗∗	∗	∗∗	∗∗∗∗∗∗
Li et al. [12]	∗∗∗	∗	∗∗	∗∗∗∗∗∗
Luo et al. [24]	∗∗∗		∗∗	∗∗∗∗∗
Oh et al. [25]	∗∗∗	∗	∗∗	∗∗∗∗∗∗
Wang et al. [27]	∗∗∗	∗	∗∗	∗∗∗∗∗∗
Luo et al. [23]	∗∗∗	∗∗	∗∗	∗∗∗∗∗∗∗
Zhang et al. [13]	∗∗∗		∗∗	∗∗∗∗∗
Feng et al. [21]	∗∗∗		∗∗	∗∗∗∗∗
Shan et al. [26]	∗∗∗		∗∗	∗∗∗∗∗
Huo et al. [22]	∗∗∗		∗∗	∗∗∗∗∗
Chen et al. [20]	∗∗∗	∗	∗∗	∗∗∗∗∗∗
Bi et al. [17]	∗∗∗	∗∗	∗∗	∗∗∗∗∗∗∗
Cai et al. [18]	∗∗∗	∗∗	∗∗	∗∗∗∗∗∗∗

^†^Selection: (1) representativeness of the exposed cohort: (a) truly representative of the average HCC patients with hypersplenism in the community (one asterisk); (b) somewhat representative of the average HCC patients with hypersplenism in the community (one asterisk); (c) selected group of users, e.g., nurses, volunteers (no asterisk); and (d) no description of the derivation of the cohort (no asterisk). (2) Selection of the nonexposed cohort: (a) drawn from the same community as the exposed cohort (one asterisk), (b) drawn from a different source (no asterisk), and (c) no description of the derivation of the nonexposed cohort (no asterisk). (3) Ascertainment of exposure to (a) secure record (e.g., surgical records) (one asterisk), (b) structured interview (one asterisk), (c) written self-report (no asterisk), and (d) no description (no asterisk). (4) Demonstration that the outcome of interest was not present at the start of the study: (a) yes (one asterisk) and (b) no (no asterisk). ^‡^Comparability: (1) Comparability of cohorts on the basis of the design or analysis: (a) study controls for liver function classification (one asterisk) and (b) study controls for any additional factor (age, gender, tumor size, tumor location, TNM stage, etc.) (one asterisk). ^§^Outcome: (1) assessment of the outcome: (a) independent blind assessment (one asterisk), (b) record linkage (one asterisk), (c) self-report (no asterisk), and (d) no description (no asterisk). (2) Was the follow-up long enough for outcomes to occur: (a) yes (select an adequate follow-up period for the outcome of interest) (one asterisk) and (b) no (no asterisk). (3) Adequacy of the follow-up of cohorts: (a) complete follow-up (all subjects accounted) (one asterisk); (b) subjects lost to follow-up unlikely to introduce bias (small number lost), >80% follow-up, or description of those lost (one asterisk); (c) follow-up rate < 80% and no description of those lost (no asterisk); and (d) no statement (no asterisk).

**Table 3:** Egger's publication bias test for effects of HS vs. HA in the treatment of patients with HCC and hypersplenism.

Outcomes	No. of trials	No. of patients	Coef. for bias	*P* for bias	95% CI for bias
5-year DFS rate	4	858	0.817	0.614	-23.332, 18.983
5-year OS rate	4	858	2.483	0.788	-32.334, 37.300
Operation time	5	751	-0.705	0.867	-13.030, 11.620
Intraoperative blood loss	8	1228	1.276	0.206	-0.927, 3.479
Intraoperative blood transfusion	6	1132	0.827	0.001	0.548, 1.105
Postoperative complications	10	1360	2.688	0.264	-2.476, 7.853
Perioperative mortality	8	1002	-1.009	0.345	-3.419, 1.402
PLT count at POD 7	5	168	5.815	0.021	1.693, 9.937
T-Bil content at POD 7	5	170	-2.663	0.383	-10.968, 5.641

Abbreviations: DFS: disease-free survival; OS: overall survival; PLT: platelet; T-Bil: total bilirubin; POD: postoperative day; Coef.: coefficient; CI: confidence interval.
